# Assessment on the Fermentation Quality and Bacterial Community of Mixed Silage of Faba Bean With Forage Wheat or Oat

**DOI:** 10.3389/fmicb.2022.875819

**Published:** 2022-05-04

**Authors:** Hongliang Li, Tairu Zeng, Zhaochang Du, Xintan Dong, Yafen Xin, Yushan Wu, Linkai Huang, Lin Liu, Bo Kang, Dongmei Jiang, Bihua Wu, Wenyu Yang, Yanhong Yan

**Affiliations:** ^1^Department of Forage Breeding and Cultivation, College of Grassland Science and Technology, Sichuan Agricultural University, Chengdu, China; ^2^Department of Crop Cultivation and Tillage, College of Agronomy, Sichuan Agricultural University, Chengdu, China; ^3^Department of Animal Science, College of Animal Science and Technology, Sichuan Agricultural University, Chengdu, China; ^4^Triticeae Research Institute, Sichuan Agricultural University, Chengdu, China

**Keywords:** faba bean, forage wheat, oat, mixed silage, fermentation quality, bacterial community

## Abstract

Faba bean (*Vicia faba L*.), although a kind of high-quality and high-yield forage, could hardly achieve a great quality of silage because of its high buffering capacity. Mixed silage of faba bean with forage wheat (*Triticum aestivum* L.) or oat (*Avena sativa* L.) at different ratios could improve the fermentation quality and bacterial community. Compared with 100% faba bean silage (BS), mixed silage improved the fermentation quality, not only increased lactic acid production and reduced pH, but reduced the production of propionic acid and ammonia nitrogen. The chemical compositions of faba bean with forage wheat (BT) mixed silage were better than that of faba bean with oat (BO) mixed silage, and that of 3:7, 5:5 (fresh matter basis) mixing ratios were better than 1:9. However, the fermentation quality of BO mixed silage was better than that of BT, and that of 3:7 mixed silage (BO30) was the best overall. Analysis of the bacterial community showed that mixed silage increased the relative abundance of lactic acid bacteria after ensiling, and the relatively higher abundance of *Lactobacillus* showed the inhibitory effects on the proliferation of *Serratia* and *Hafnia_Obesumbacterium*, so that it alleviated their negative effects on silage and stabilized the fermentation quality. This present study exhibited that mixed silage of faba bean with forage wheat or oat not only had significant effects on chemical compositions and fermentation quality of materials but modified bacterial community so that improved the fermentation quality effectively. The mixed silage of 30% faba bean with 70% oat (BO30) is recommended in the faba bean mixed silage.

## Introduction

Ensiling is an effective technique for the long-term preservation of forage nutrients, in which microorganisms ferment sugars into acids, reduce the pH under anaerobic conditions, and ultimately inhibit the proliferation of undesirable microorganisms (Fabiszewska et al., 2019). The utilization of silage is contributed to overcoming the discrepancy between livestock production and the seasonal imbalance of available forage (Wright et al., 2000), so provides a guarantee for animal husbandry. In addition to higher protein content compared to grass silage, legume silage also has higher intakes and animal production than grass silage of comparable digestibility, which could enhance the growth rate and milk yield (Dewhurst et al., 2003, 2009). Thus, legume silage has a high feeding value. However, legume forage could hardly achieve high-quality silage in natural fermentation conditions (without any additive or strain inoculates). The most common measurements used for evaluating silage fermentation include pH, organic acids, ammonia nitrogen (NH_3_-N), and the various microbial community (Kung Jr et al., 2018). The high buffering capacity of legume forages (Colombini et al., 2007; Borreani et al., 2009), usually obstructs the reduction of pH, making pH hardly reach the general standard of 4.2 (Wang et al., 2017), such as alfalfa silage and soybean silage (Filya et al., 2007; Ni et al., 2018). The subsequent activities of some undesirable bacteria may lead to the degradation of protein and the production of NH_3_-N, ultimately resulting in poor fermentation quality. Gramineous forage contains sufficient substrate of fermentation-sugars, and the lower buffering capacity also makes its fermentation quality generally better even in natural fermentation conditions, such as corn silage, sorghum silage, and napier silage (Aksu et al., 2004; Zhang et al., 2011; Alhaag et al., 2019). However, grass silage is high-energy and low-protein forage, which is better used in combination with high-protein forage such as alfalfa to satisfy the nutritional requirements of livestock (Allen et al., 2003).

The success of the silage fermentation process is affected by various factors, such as the forage features during the harvesting period, climatic conditions, silage facilities, epiphytic microbial community of forage, and so on (McEniry et al., 2008; Zhang et al., 2016). Microorganisms especially play a vital role, and the other factors are aimed at providing excellent conditions for microorganisms which conducive to silage fermentation. The studies of microbial community during silage not only perceived the principle of ensiling but also established the key role of bacteria in the whole fermentation process (McEniry et al., 2008; Naoki and Yuji, 2008). Lactic acid bacteria (LAB), for example, *Lactobacillus, Lactococcus, Leuconostoc, Enterococcus, Paralactobacillus, Pediococcus*, and *Weissella* are beneficial to silage fermentation, while some enterobacteria, clostridia, acetic acid bacteria, and aerobic bacteria are undesirable to silage fermentation (Moon and Ely, 1979; Spoelstra et al., 1988; Heron et al., 1993; Brusetti et al., 2006; Parvin et al., 2010; Muck, 2013). Therefore, the fermentation quality is directly related to the bacterial community.

The shortage of forage and the discrepancy of forage in winter and spring in China have hindered the development of herbivorous animal husbandry and become a weakness for the development of the whole animal husbandry (Li et al., 2015), so it is very important to improve the utilization of silage and expand the source of silage raw materials. Mixed silage has been found to improve silage quality and enhance the stability of the fermentation system compared to sole silage (Larsen et al., 2017; Jiang et al., 2018). Soybean with corn mixed silage has been widely studied, the results showed that compared to legume sole silage, mixed silage improved fermentation quality, and had higher energy than grass sole silage (Ni et al., 2018; Zeng et al., 2020). Thus, it can be concluded that mixed silage enhanced the benefits and reduced the shortcomings of sole silage.

Faba bean (*Vicia faba* L.) is considered the third most essential forage grain and winter legume (Mihailovic et al., 2005) and is extensively grown around the world. The yield of faba bean could reach 6–7 t/ha (Mínguez and Rubiales, 2021), and it is rich in protein and powerful in biological nitrogen fixation (Turpin et al., 2002). As a green manure, it could also control the growth of weeds (Álvarez-Iglesias et al., 2018). Thus, the planting of faba bean possesses economic and environmental benefits, and it is an excellent raw material for silage. Forage wheat (*Triticum aestivum* L.) is generally considered the source of calories for animal feed production because of its high content of starch (Shewry, 2009). Oat (*Avena sativa* L.) has a relatively high feeding value as it is high yield (Baron et al., 1992) and has great animal intake (Hingston and Christensen, 1982). There are also a lot of relevant studies on oat reported, such as the effects of wilting (Gomes et al., 2019), inoculation (Romero et al., 2017), and microbial community (Wang et al., 2020). Moreover, as winter crops, the growth periods of those two plants are similar to that of faba bean, which provides a proper condition for creating faba bean mixed silage. But unfortunately, the silage quality and the optimal proportion of the mixture still remain unknown. At the same time, the study of the silage bacterial community is also necessary since the epiphytic microbial species of different raw materials are different (Ramírez-Vega et al., 2020).

The purposes of this study are as follows:

1, to investigate the optimal ratios of faba bean with forage wheat or oat mixed silage;
2, to reveal the connections between bacterial community and silage quality.

And the study would provide a theoretical basis for the mixed silage of legumes and grasses in practical productions.

## Materials and Methods

### Materials and Silage Treatments

Faba bean (Yundou-147), oat (Magnum) and forage wheat (Chuannong-01) were sown at the Chongzhou experimental base of Sichuan Agricultural University, Chengdu, China (30°13' N, 103°23' E) on 23 October 2019, and were harvested on 30 April 2020 with a 5 cm stubble height, during which faba bean was at the terminal seed filling period, oat was at milk stage and forage wheat was at waxy ripening stage. Mowed forages were chopped into theoretical lengths of 10-20 mm. All silage treatments based on fresh matter (FM) are as follows: (1) 100% faba bean (BS); (2) 100% forage wheat (TS); (3) 100% oat (OS); (4) 10% faba bean and 90% forage wheat mixed uniformly (BT10); (5) 30% faba bean and 70% forage wheat mixed uniformly (BT30); (6) 50% faba bean and 50% forage wheat mixed uniformly (BT50); (7) 10% faba bean and 90% oat mixed uniformly (BO10); (8) 30% faba bean and 70% oat mixed uniformly (BO30); (9) 50% faba bean and 50% oat mixed uniformly (BO50). Samples of 300 g, in triplicate, were packed into polyethylene plastic bags (dimensions 25 cm × 35 cm, China), then were vacuum sealed. A total of 27 bags were preserved at room temperature (25–30°C). The chemical compositions, fermentation quality, and bacterial community were analyzed for raw materials and samples after 60 days of ensiling.

### Measurement of Chemical Compositions and Fermentation Quality

All silage samples were oven-dried at 65°C for 72 h for the dry matter (DM) measurement; chemical analysis was done by using a 1 mm Wiley mill screen; the content of water-soluble carbohydrate (WSC) was assayed by the thracenone-sulphuric acid method (AOAC, 1990); neutral detergent fiber (NDF) and acid detergent fiber (ADF) contents were measured based on the method of Van Soest et al. (1991). The Kjeldahl method was used to determine the crude protein (CP) content (AOAC, 1990), and NH_3_-N as a percentage of total nitrogen (TN) content was assayed as Broderick and Kang (1980) described. Then 20 g samples were put in a blender, homogenized with 180 mL sterilized water for 1 min, and filtered *via* medical gauze with four layers. After that, the pH of filtrate was detected with a pH meter (PHSJ-5; LEICI, Shanghai, China). Lactic acid (LA), acetic acid (AA), propionic acid (PA), and butyric acid (BA) were determined by high-performance liquid chromatography (Shimadzu, Kyoto, Japan), which was fitted with a UV detector (210 nm) and a column (KC-811; Zeng et al., 2020).

### Microbial Population Counting

Similar to the study of Yan et al. (2019), three common microorganisms in silage raw material were detected. Samples of 20 g were put in a table concentrator, mixed thoroughly with 180 mL of sterile saline (0.85% NaCl) for 1 h, and filtered *via* medical gauze with four layers, then serially diluted from 10^−1^ to 10^−7^ colony-forming units cfu/mL. The LAB was counted by De Man, Rogosa, and Sharpe agar; enterobacter was counted by Violet Red Bole Agar; yeasts and molds were counted by Potato Dextrose Agar (Difco, Land Bridge, Beijing, China).

### Bacterial Community Analysis

The TIANamp Bacteria DNA isolation kit (DP302-02, Tiangen, Beijing, China) was used to extract total genomic DNA. The extracted DNA was depurated and recovered by the DNA kit column (DP214-02, Tiangen, Beijing, China) before being eluted in nuclease-free water. The quality of the extracted DNA was detected by 1% agarose gels electrophoresis and spectrophotometry (optical density at 260/280 nm ratio), and qualified DNA samples were stored at −20°C for further analysis.

A total of 16S rRNA genus of distinct regions (V4) were amplified by using the specific primers 515F (5′-GTGCCAGCMGCCGCGGTAA-3′) and 806R (5′-GGACTACHVGGGTWTCTAAT-3′) with the barcode. Samples with a clear main strip of 400–450 bp were chosen for the next analysis. PCR products were mixed equally and were detected by electrophoresis with agarose gel of 2% concentration according to the concentration of PCR products. The target bands were recovered by the gel recovery kit (Qiagen). Truseq^®^ DNA PCR free sample preparation kit was used for sequencing library construction. The constructed sequencing library was quantified by Qubit and Q-PCR, and novaseq6000 was used for sequencing.

Next-generation sequencing reads were assembled by using FLASH (V1.2.7, http://ccb.jhu.edu/software/FLASH/). Raw tags data was obtained by splicing the reads of each sample, and high-quality tags data was generated by removing low-quality reads according to the QIIME quality control process (V1.9.1, http://qiime.org/scripts/split_libraries_fastq.html). Tags sequence was compared with the species annotation database (https://github.com/torognes/vsearch/) to detect the chimeric sequence, and finally the chimeric was removed sequence to obtain the final effective tags. All effective tags were performed by Uparse software (v7.0.1001, http://www.drive5.com/uparse/). A 97% similarity was used to define operational taxonomic units (OTUs). Species annotation of OTUs sequences was performed by the Mothur method and SSUrRNA database of SILVA138 (http://www.arb-silva.de/). Alpha diversity metrics including observed-species, chao1, Shannon, Simpson, abundance-based coverage estimator (ACE), and good coverage were calculated with Qiime software (Version 1.9.1) and analyzed by R software (Version 2.15.3). The relative abundance bar-plot of bacteria community, petal diagram analysis, and Spearman correlation heatmap were also completed by R software. The linear discriminant analysis (LDA) effect size (LEfSe) was completed by LEfSe software. The raw sequence data had been uploaded to the sequence read archive (SRA) of the NCBI database under the accession number PRJNA778801.

### Statistical Analyses

Microbial populations of raw materials and silage were estimated as cfu/g FM and were log-transformed prior to statistical analysis. All data were presented as an average of replicate tests and were analyzed by one-way and two-way analysis of variance (SPSS 19.0 Chicago, IL, USA). And the least significant difference (LSD) tests were at *P* < 0.05 using the SAS program version 9.1 (SAS Institute, Cary, NC).

## Results

### Characteristics of Raw Materials

The chemical composition and microbial population in raw materials are shown in Table 1. The DM content of fresh forage wheat (FT) was 402.7 g/kg FM, much higher than that of fresh faba bean (FB) and fresh oat (FO) (*P* < 0.05). FB had the highest CP content (188.3 g/kg DM), while that of FO and FT were only 61.8 and 82.1 g/kg DM, respectively. Among raw materials, the contents of NDF and ADF ranged from 428.5 to 544.5 g/kg DM and 211.0 to 325.7 g/kg DM, respectively, and both were the highest in FB (*P* < 0.05). The NDF content of FO was lower than that of FT (*P* < 0.05), while their ADF content was of a comparable level. The WSC content of FB was 126.5 g/kg DM, lower than that of FO and FT (182.4 and 172.2 g/kg DM, respectively; *P* < 0.05). The microbial counting presented that the epiphytic LAB number of FO was 4.76 log_10_ cfu/g FM, and that of FB and FT were 3.67 and 3.85 log_10_ cfu/g FM, respectively. And the population of yeasts was the smallest in FO (3.15 log_10_ cfu/g FM), and it was almost the same in FT and FB. The number of enterobacter in FB was 2.59 log_10_ cfu/g FM, while it was <2.0 log_10_ cfu/g FM in both FO and FT. Molds were not detected in all raw materials.

**Table 1:** Chemical composition and microbial population in fresh raw materials.

**Items**	**FB**	**FO**	**FT**	**SEM**
DM (g/kg FM)	227.8^c^	258.7^b^	402.7^a^	2.70
CP (g/kg DM)	188.3^a^	61.8^c^	82.1^b^	1.98
NDF (g/kg DM)	544.5^a^	428.5^c^	459.1^b^	1.77
ADF (g/kg DM)	325.7^a^	211.0^b^	221.5^b^	1.88
WSC (g/kg DM)	126.5^c^	182.4^a^	172.2^b^	0.87
LAB (log_10_ cfu/g FM)	3.67^b^	4.76^a^	3.85^b^	0.18
Enterobacter (log_10_ cfu/g FM)	2.59	<2.00	<2.00	-
Yeasts (log_10_ cfu/g FM)	3.70^a^	3.15^b^	3.71^a^	0.10
Molds (log_10_ cfu/g FM)	<2.00	<2.00	<2.00	-

*FM, fresh matter; DM, dry matter; LAB, lactic acid bacteria; CP, crude protein; NDF, neutral detergent fiber; ADF, acid detergent fiber; WSC, water-soluble carbohydrate; cfu, colony-forming unit; FB, fresh faba bean; FO, fresh oat; FT, fresh forage wheat; SEM, standard error of means; Data are the means of three samples; Values followed by the same lowercase letters indicate no significant difference (P < 0.05)*.

### Chemical Composition and Fermentation Quality After Ensiling

Chemical composition after 60 days of ensiling is presented in Table 2. The grass species (*P* < 0.01), mixing ratios (*P* < 0.01), and their interactions (*P* < 0.01) influenced DM content. The DM content of BT mixed silage was higher than that of BO (*P* < 0.05), while that of 1:9 and 3:7 mixing ratios were higher than that of 5:5 (*P* < 0.05). Different grass species (*P* < 0.01), mixing ratios (*P* < 0.01) and their interactions (*P* = 0.012) had an effect on CP content. BT mixed silage had higher CP content than BO (*P* < 0.05). The CP content increased with the increasing proportion of faba bean in mixed silage, in which that of the 3:7 and 5:5 mixing ratios were higher than that of 1:9 (*P* < 0.05), but there was no difference between them (*P* < 0.05). The NDF content was affected by grass species (*P* < 0.01) and mixing ratios (*P* < 0.01). BT mixed silage had higher NDF content than BO (*P* < 0.05), and there were significant differences among different mixing ratios (*P* < 0.05). The ADF content was affected by grass species (*P* = 0.04) and mixing ratios (*P* < 0.01). BT mixed silage had higher ADF content than BO, and that of the 3:7 and 5:5 mixing ratios were higher than that of 1:9 (*P* < 0.05). Grass species (*P* < 0.01) and mixing ratios (*P* = 0.04) had an influence on WSC content, in which the WSC content of BO mixed silage was lower than that of BT (*P* < 0.05).

**Table 2:** Chemical composition after ensiling.

**Items**	**BS**	**OS**	**TS**	**Ratio**	**BO**	**BT**	**Mean**	**SEM**	***P*** **-value**		
									**Species**	**Ratio**	**S×R**
DM (g/kg FM)	215.0	244.6	381.2	10	257.4^Ba^	375.1^Aa^	316.3^a^	11.85	<0.01	<0.01	<0.01
				30	246.5^Ba^	351.8^Ab^	299.2^a^				
				50	241.8^Ba^	309.9^Ac^	275.9^b^				
Mean					247.6^B^	354.5^A^					
CP (g/kg DM)	161.9	51.9	81.4		70.3^Bc^	88.0^Ac^	79.2^b^	5.86	<0.01	<0.01	0.012
					89.0^Bb^	107.0^Ab^	98.0^a^				
					98.5^Ba^	119.8^Aa^	109.2^a^				
Mean					77.4^B^	99.0^A^					
NDF (g/kg DM)	543.3	421.1	450.8		435.2	453.7	444.5^c^	7.58	<0.01	<0.01	0.39
					441.5	488.3	464.9^b^				
					467.7	509.0	488.4^a^				
Mean					441.4^B^	475.5^A^					
ADF (g/kg DM)	317.1	201.8	216.0		202.6	218.4	210.5^b^	6.56	0.04	<0.01	0.63
					232.6	235.9	234.3^a^				
					245.4	249.8	247.5^a^				
Mean					220.6^B^	230.0^A^					
WSC (g/kg DM)	58.2	16.8	24.5		26.8	31.4	29.1^a^	2.42	<0.01	0.04	0.27
					22.6	33.2	27.9^b^				
					21.8	34.8	28.3^b^				
Mean					22.0^B^	31.0^A^					

*FM, fresh matter; DM, dry matter; CP, crude protein; NDF, neutral detergent fiber; ADF, acid detergent fiber; WSC, water-soluble carbohydrate; SEM, standard error of means. BS, 100% faba bean silage; OS, 100% oat silage; TS, 100% forage wheat silage; BO10, 10% faba bean with 90% oat mixed silage; BO30, 30% faba bean with 70% oat mixed silage; BO50, 50% faba bean with 50% oat mixed silage; BT10, 10% faba bean with 90% forage wheat mixed silage; BT30, 30% faba bean with 70% forage wheat mixed silage; BT50, 50% faba bean with 50% forage wheat mixed silage; Data are the means of three samples; Values followed by the same lowercase letters indicates no significant difference across different mixing ratios in the same grass (P < 0.05, vertical comparison); Values followed by the same capital letters indicates no significant difference across different grass in the same mixing ratios (P < 0.05, horizontal comparison)*.

Organic acids, pH, and NH_3_-N after 60 days of ensiling were determined and are shown in Table 3. The grass species had a significant effect on pH value (*P* < 0.01). BS silage had a pH of 4.75, while the pH of BO and BT mixed silage was between 4.1 and 4.3, and that of BO was lower than BT (*P* < 0.05). The LA content was affected by grass species (*P* < 0.01), mixing ratios (*P* < 0.01) and their interactions (*P* < 0.01). BS had the lowest LA content (19.07 g/kg DM), while that in mixed silage was increased. The LA content of BO mixed silage was higher than that of BT (*P* < 0.05), while that of 1:9 and 3:7 mixing ratios were higher than 5:5 (*P* < 0.05). The same with LA content, AA content was also influenced by grass species (*P* < 0.01), mixing ratios (*P* < 0.01) and their interactions (*P* < 0.01), and that of BO mixed silage was higher than that of BT (*P* < 0.05), while 5:5 mixing ratio had the lowest AA content (*P* < 0.05). Grass species had an effect on PA content (*P* = 0.023), and that of BT mixed silage was higher than that of BO (*P* < 0.05). Grass species also had an effect on NH_3_-N content (*P* < 0.01). The NH_3_-N content in BS was 8.44% TN, and that of mixed silage ranged from 2.85 to 3.57% TN, in which the NH_3_-N content of BT mixed silage was higher than that of BO (*P* < 0.05).

**Table 3:** Fermentation quality after ensiling.

**Items**	**BS**	**OS**	**TS**	**Ratio**	**BO**	**BT**	**Mean**	**SEM**	***P*** **-value**		
									**Species**	**Ratio**	**S×R**
pH	4.21	4.21	4.24	10	4.16	4.24	4.20	0.35	<0.01	0.69	0.52
				30	4.16	4.25	4.21				
				50	4.17	4.28	4.23				
Mean					4.18^B^	4.26^A^					
LA (g/kg DM)	19.07	35.24	28.87		45.42^Ab^	36.59^Ba^	41.00^ab^	1.97	<0.01	<0.01	<0.01
					55.80^Aa^	32.23^Bb^	44.02^a^				
					37.34^Ac^	29.81^Bb^	33.58^b^				
Mean					43.45^A^	31.88^B^					
AA (g/kg DM)	16.19	12.79	10.57		12.51^Ab^	13.33^Aa^	12.92^a^	0.82	<0.01	<0.01	<0.01
					17.37^Aa^	12.29^Ba^	14.83^a^				
					10.52^Ac^	9.83^Ab^	10.17^b^				
Mean					13.30^A^	11.50^B^					
PA (g/kg DM)	14.78	8.67	9.73		8.80	9.46	9.13	0.35	0.023	0.33	0.59
					9.54	9.81	9.68				
					9.32	9.63	9.48				
Mean					9.08^B^	9.66^A^					
BA (g/kg DM)	1.65	-	-		-	-	-	-	-	-	-
					-	-	-				
					-	-	-				
Mean					-	-					
NH_3_-N (% TN)	8.44	3.18	3.94		2.85	3.48	3.17	0.33	<0.01	0.17	0.69
					2.78	3.49	3.14				
					3.26	3.57	3.42				
Mean					3.02^B^	3.62^A^					

*DM, dry matter; LA, lactic acid; AA, acetic acid; PA, propionic acid; BA, butyrate acid; NH_3_-N, ammonia nitrogen; TN, total nitrogen; SEM, standard error of means. BS, 100% faba bean silage; OS, 100% oat silage; TS, 100% forage wheat silage; BO10, 10% faba bean with 90% oat mixed silage; BO30, 30% faba bean with 70% oat mixed silage; BO50, 50% faba bean with 50% oat mixed silage; BT10, 10% faba bean with 90% forage wheat mixed silage; BT30, 30% faba bean with 70% forage wheat mixed silage; BT50, 50% faba bean with 50% forage wheat mixed silage; Data are the means of three samples; Values followed by the same lowercase letters indicate no significant difference across different mixing ratios in the same grass (P < 0.05, vertical comparison); Values followed by the same capital letters indicate no significant difference across different grass in the same mixing ratios (P < 0.05, horizontal comparison)*.

The microbial population after 60 days of ensiling is presented in Table 4. Enterobacter was only detected in BS, BO50, and BT50, in which the number in BS was 4.21 log_10_ cfu/g FM, and 2.21 and 3.13 log_10_ cfu/g FM in BO50 and BT50, respectively. The number of LAB was affected by grass species (*P* < 0.01), mixing ratios (*P* < 0.01), and their interactions (*P* < 0.01), in which the number of LAB in BO mixed silage was higher than that in BT (*P* < 0.05), while 1:9 and 3:7 mixing ratios had more LAB than 5:5 (*P* < 0.05). Besides, molds were undetected in all silage, and yeasts were only detected in BS (2.56 log_10_ cfu/g FM).

**Table 4:** Microbial population after ensiling.

**Items**	**BS**	**OS**	**TS**	**Ratio**	**BO**	**BT**	**Mean**	**SEM**	***P*** **-value**		
									**Species**	**Ratio**	**S×R**
Enterobacter (log_10_ cfu/g FM)	4.21	<2.00	<2.00	10	<2.00	<2.00	-	-	-	-	-
				30	<2.00	<2.00	-				
				50	2.21	3.31	-				
Mean					-	-					
LAB (log_10_ cfu/g FM)	3.92	5.22	4.54		5.41^Aa^	4.55^Ba^	4.98^a^				
					5.36^Aa^	4.47^Bb^	4.92^a^	0.16	<0.01	<0.01	<0.01
					4.70^Ab^	4.53^Aab^	4.62^b^				
					5.17^A^	4.52^B^					
Molds (log_10_ cfu/g FM)	3.92	<2.00	<2.00		<2.00	<2.00	-	-	-	-	-
					<2.00	<2.00	-				
					<2.00	<2.00	-				
Mean					-	-	-				
Yeast (log_10_ cfu/g FM)	2.56	<2.00	<2.00		<2.00	<2.00	-	-	-	-	-
					<2.00	<2.00	-				
					<2.00	<2.00	-				
Mean					-	-	-				

*FM, fresh matter; LAB, lactic acid bacteria; SEM, standard error of means; cfu, colony-forming unit; BS, 100% faba bean silage; OS, 100% oat silage; TS, 100% forage wheat silage; BO10, 10% faba bean with 90% oat mixed silage; BO30, 30% faba bean with 70% oat mixed silage; BO50, 50% faba bean with 50% oat mixed silage; BT10, 10% faba bean with 90% forage wheat mixed silage; BT30, 30% faba bean with 70% forage wheat mixed silage; BT50, 50% faba bean with 50% forage wheat mixed silage; Data are the means of three samples; Values followed by the same lowercase letters indicate no significant difference across different mixing ratios in the same grass (P < 0.05, vertical comparison); Values followed by the same capital letters indicate no significant difference across different grass in the same mixing ratios (P < 0.05, horizontal comparison)*.

In conclusion, grass species and mixing ratios had effects on the chemical compositions, as well as the content of pH, LA, AA, and NH_3_-N contents in mixed silage. The chemical compositions of BT mixed silage were better than that of BO, and 3:7 and 5:5 mixing ratios were better than 1:9. But the fermentation quality of BO mixed silage was better than those of BT, and the 3:7 mixing ratio was the best overall. Meanwhile, compared with the typically recommended values of common fermentation end products in silage (Kung Jr et al., 2018), it was found that the fermentation quality of mixed silage basically reached the qualified quality.

### Bacterial Community of Silage

**Figure 1:**
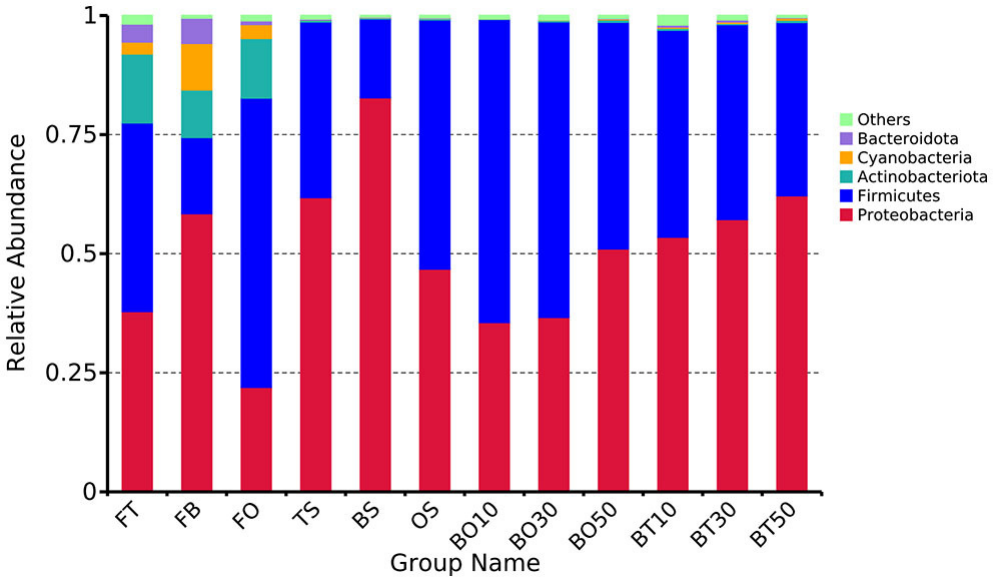
Relative abundance of bacteria at the phylum level. FT, fresh forage wheat; FB, fresh faba bean; FO, fresh oat; BS, 100% faba bean silage; OS, 100% oat silage; TS, 100% forage wheat silage; BO10, 10% faba bean with 90% oat mixed silage; BO30, 30% faba bean with 70% oat mixed silage; BO50, 50% faba bean with 50% oat mixed silage; BT10, 10% faba bean with 90% forage wheat mixed silage; BT30, 30% faba bean with 70% forage wheat mixed silage; BT50, 50% faba bean with 50% forage wheat mixed silage.

**Figure 2:**
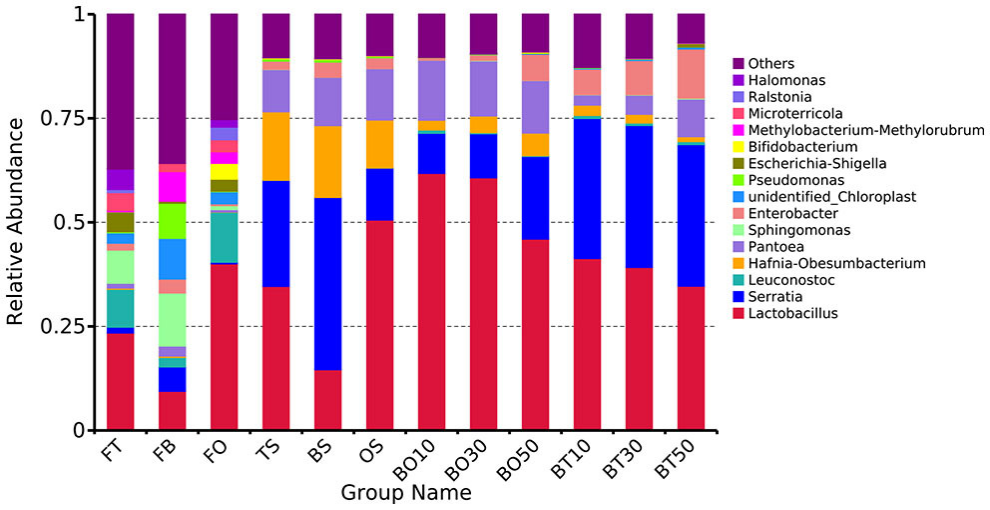
Relative abundance of bacteria at the genus level. FT, fresh forage wheat; FB, fresh faba bean; FO, fresh oat; BS, 100% faba bean silage; OS, 100% oat silage; TS, 100% forage wheat silage; BO10, 10% faba bean with 90% oat mixed silage; BO30, 30% faba bean with 70% oat mixed silage; BO50, 50% faba bean with 50% oat mixed silage; BT10, 10% faba bean with 90% forage wheat mixed silage; BT30, 30% faba bean with 70% forage wheat mixed silage; BT50, 50% faba bean with 50% forage wheat mixed silage.

**Figure 3:**
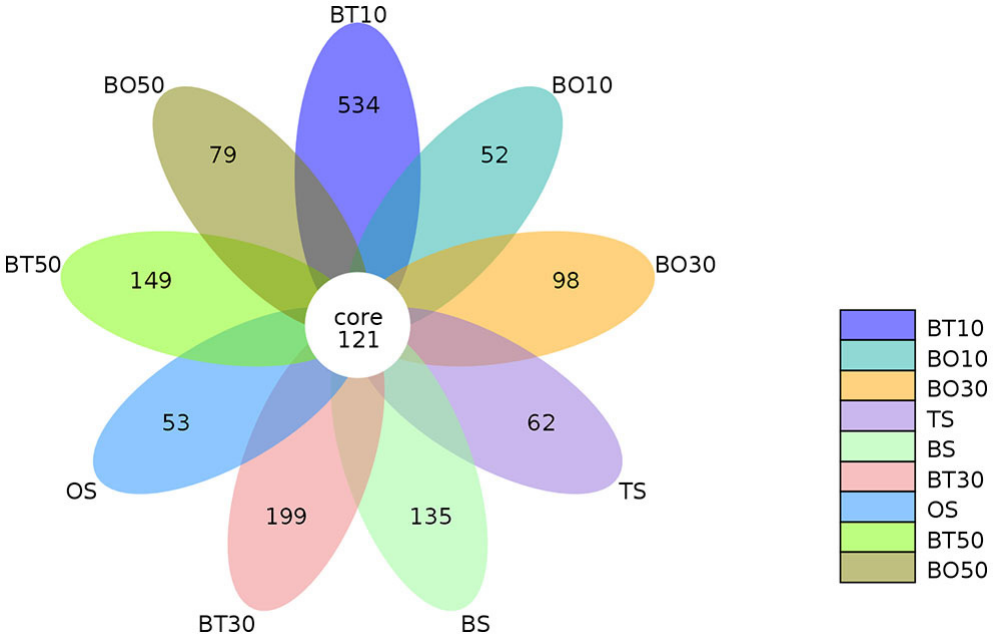
Petal diagram analysis of operational taxonomic units. FT, fresh forage wheat; FB, fresh faba bean; FO, fresh oat; BS, 100% faba bean silage; OS, 100% oat silage; TS, 100% forage wheat silage; BO10, 10% faba bean with 90% oat mixed silage; BO30, 30% faba bean with 70% oat mixed silage; BO50, 50% faba bean with 50% oat mixed silage; BT10, 10% faba bean with 90% forage wheat mixed silage; BT30, 30% faba bean with 70% forage wheat mixed silage; BT50, 50% faba bean with 50% forage wheat mixed silage.

**Figure 4:**
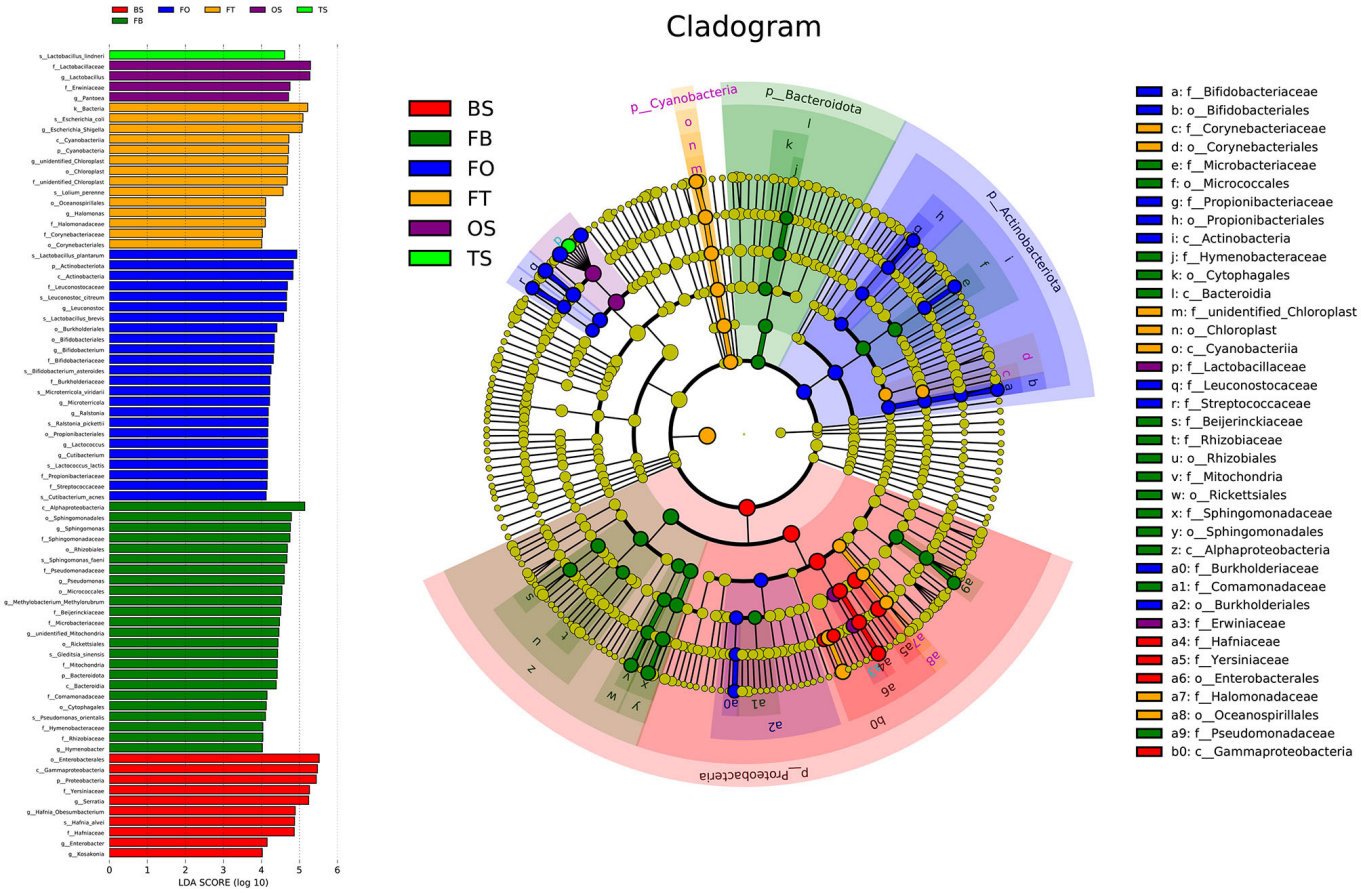
Comparison of bacterial variations of faba bean, oat, and forage wheat before and after ensiling using the LEfSe online tool. FT, fresh forage wheat; FB, fresh faba bean; FO, fresh oat; TS, 100% forage wheat silage; OS, 100% oat silage; BS, 100% faba bean silage.

**Figure 5:**
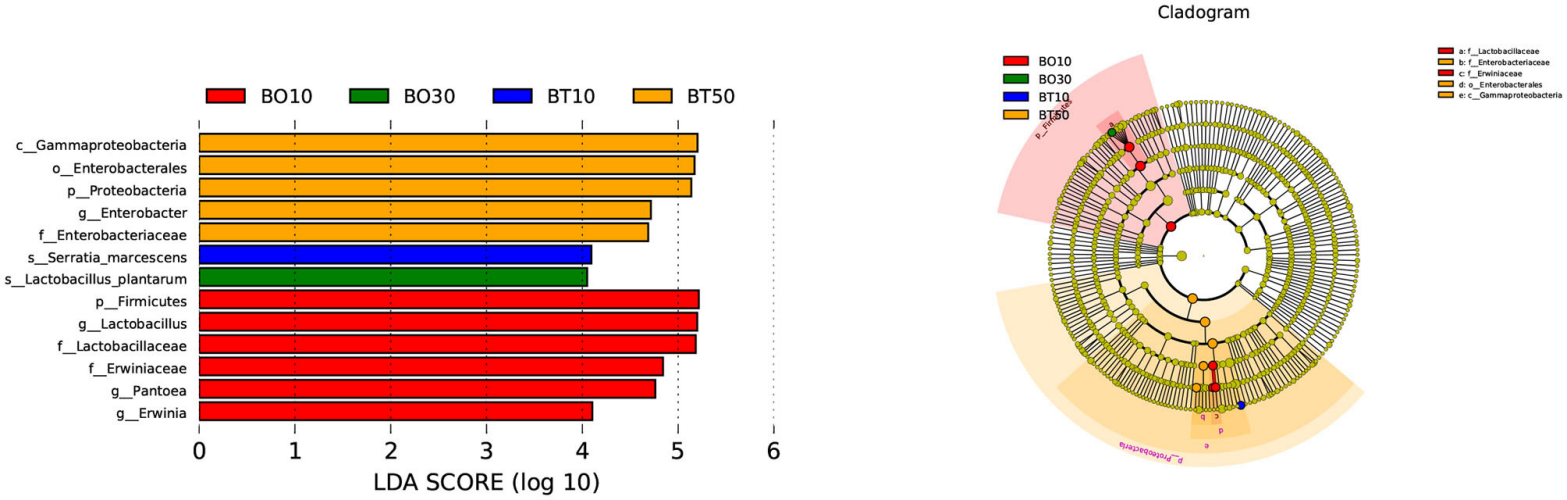
Comparison of bacterial variations in mixed silage using the LEfSe online tool. BO10, 10% faba bean with 90% oat mixed silage; BO30, 30% faba bean with 70% oat mixed silage; BT10, 10% faba bean with 90% forage wheat mixed silage; BT50, 50% faba bean with 50% forage wheat mixed silage.

A total of 22,862,843 raw reads were processed. The bacterial alpha diversity indexes of silage including Shannon, Simpson, Ace, and Chao 1 indices of all silage are shown in Table 5. The Good's coverage value of all silage treatments was around 0.99, indicating that the sequencing had adequately captured most of the bacterial community. The bacterial diversity decreased in sole silage treatments compared to their raw materials, and the Shannon index of bacterial diversity was observed to be lower in BO mixed silage than in BT. Proteobacteria, Firmicutes, Actinobacteria, Cyanobacteria, and Bacteroidetes took advantage of all raw materials, whereas Proteobacteria and Firmicutes completely dominated the entire bacterial community after ensiling (Figure 1). At the genus level, mixed silage had a higher relative abundance of *Lactobacillu* compared to BS, and that of BO silage was higher than that of BT silage (56.24 vs. 38.42%). *Leuconostoc* was found in a small visible relative abundance in mixed silage, while *Serratia* increased greatly after ensiling, and the relative abundance of *Serratia* in BT mixed silage was higher than that of BO mixed silage (13.3 vs. 33.9%). Meanwhile, *Hafnia_Obesumbacterium*, which was almost absent in raw materials, was presented after ensiling. The relative abundance of *Hafnia_Obesumbacterium* in sole silage was higher than that of mixed silage, and that of BT mixed silage was lower than that of BO (1.90 vs. 3.93%) (Figure 2). Petal diagram analysis of OTUs showed that all silage treatments contained 121 common OTUs (Figure 3). The unique OTUs of BS were higher than that of OS and TS, while that of BO mixed silage was lower than that of BT mixed silage. The linear discriminant analysis (LDA) effect size (LEfSe) method was used to compare the bacterial variations of faba bean, oat, and forage wheat before and after ensiling (LDA score > 4.0; Figure 4). The raw materials had a wide variety of bacteria, but the types of bacteria significantly decreased after ensiling. *Hafnia_Obesumbacterium, Serratia*, and *enterobacter* became the abundant genus in BS compared to FB. *Lactobacillus* increased in OS compared to FO, whose LDA score was over 5.0. *Lactobacillus_lindneri* became the abundant species in TS. The comparison of bacterial variations in mixed silage was shown in Figure 5. *Lactobacillus* was abundant at the genus level in BO10. *Lactobacillus_plantarum* was the abundant species in BO30, and *Serratia_marcescens* became the abundant species in BT10. *Enterobacter* was the abundant genus in BT50, and the LDA score of no bacteria in BO50 and BT30 exceeded 4.0.

**Table 5:** General information of bacterial diversity.

**Silage**	**Observed species**	**Shannon index**	**Simpson index**	**ACE index**	**Chao 1 index**	**Coverage**
FB	453	5.77	0.96	484.49	485.18	0.99
FO	497	4.85	0.90	559.58	553.11	0.99
FT	427	4.85	0.92	470.37	465.62	0.99
BS	418	3.19	0.78	316.86	308.89	0.99
OS	325	3.43	0.80	368.48	355.98	0.99
TS	281	3.57	0.83	376.64	363.50	0.99
BO10	253	3.93	0.89	376.13	415.52	0.99
BO30	393	3.80	0.85	444.04	431.20	0.99
BO50	361	3.58	0.81	439.59	428.63	0.99
BT10	377	4.21	0.86	335.55	316.00	0.99
BT30	512	4.10	0.86	592.10	579.01	0.99
BT50	324	3.80	0.85	406.23	391.87	0.99

*FB, fresh faba bean; FO, fresh oat; FT, fresh forage wheat; BS, 100% faba bean silage; OS, 100% oat silage; TS, 100% forage wheat silage; BO10, 10% faba bean with 90% oat mixed silage; BO30, 30% faba bean with 70% oat mixed silage; BO50, 50% faba bean with 50% oat mixed silage; BT10, 10% faba bean with 90% forage wheat mixed silage; BT30, 30% faba bean with 70% forage wheat mixed silage; BT50, 50% faba bean with 50% forage wheat mixed silage; Data are the means of three samples*.

### Association Analysis Between Fermentation Quality and Bacterial Community

**Figure 6:**
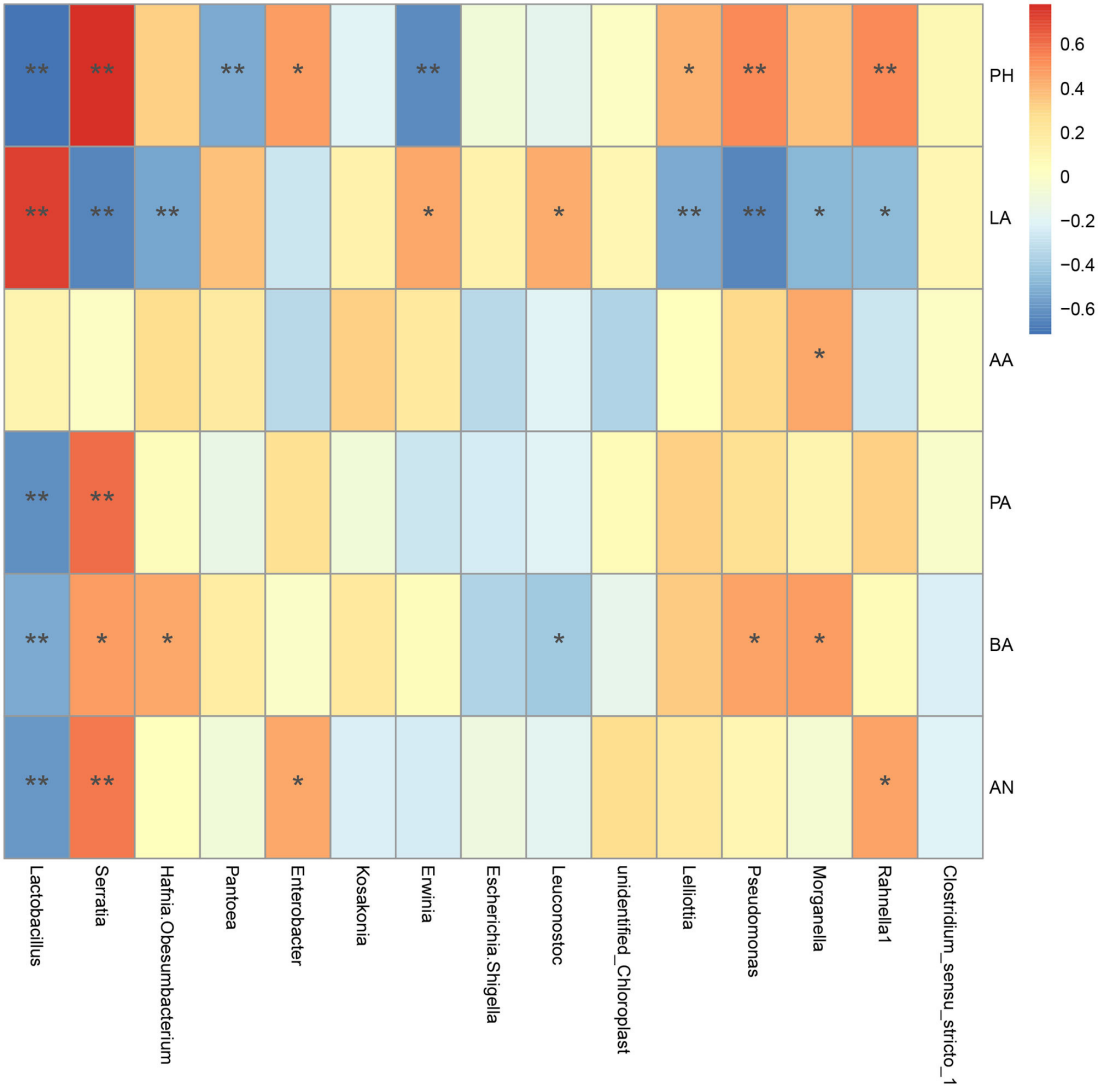
Spearman correlation heatmap of bacterial community with fermentation characteristics at the genus level. A positive correlation was indicated by red color, and negative correlation was indicated by blue color. “*” and “**” represent *P* < 0.05 and *P* < 0.01, respectively. LA, lactic acid; AA, acetic acid; PA, propionic acid; BA, butyric acid; AN, Ammonia nitrogen.

The relevance between fermentation quality and bacterial communities was assayed and is presented in Figure 6. The pH was positively associated with *Serratia* and *Pseudomonas* (*P* < 0.01), and negatively related to *Lactobacillus, Pantoea*, and *Erwinia* (*P* < 0.01). The LA content was positively correlated to *Lactobacillus* (*P* < 0.01), *Leuconostoc*, and *Erwinia* (*P* < 0.05), and negatively correlated with *Serratia, Hafnia_Obesumbacterium, Lelliottia*, and *Pseudomonas* (*P* < 0.01). The AA content was positively correlated with *Morganella* (*P* < 0.05). The PA content was positively associated with *Serratia* (*P* < 0.01), while it was negatively related to *Lactobacillus* (*P* < 0.01). The BA content was positively corresponded to *Serratia, Hafnia_Obesumbacterium, Pseudomonas* and *Morganella* (*P* < 0.05), and negatively correlated with *Lactobacillus* (*P* < 0.01) and *Leuconostoc* (*P* < 0.05). And the NH_3_-N content had a negative relationship with *Lactobacillus* (*P* < 0.01), and a positive relationship with *Serratia* (*P* < 0.01) and *enterobacter* (*P* < 0.05). It could be seen that the beneficial bacteria in silage were mainly *Lactobacillus* and *Leuconostoc*, while the undesirable bacteria were mainly *Serratia, hafnia_ Obesumbacterium, Morganella*, and *Pseudomonas* in the current study.

## Discussion

### Effect of Mixed Silage of Faba Bean With Forage Wheat or Oat on Silage Quality

The DM content of faba bean raw material (FB) was relatively low in this study (227.8 g/kg). Borreani et al. (2009) also harvested the faba bean during the same period, with a result of 237.0 g/kg DM content, similar to the current study. This indicates that the faba bean harvested in this period had high moisture and may affect the fermentation quality, so it is worth trying to wither prior to ensiling. Interestingly, the DM content of FO is lower than that of FT, but the pH was lower and LA content was higher in BO mixed silage than that of BT mixed silage. The yeasts in FT were higher than that in FO, the epiphytic LAB of FT was lower, and higher CP content also led to higher buffering capacity. These factors may jointly result in the poorer fermentation of BT mixed silage compared to BO. The yeasts population of all silage was relatively low, this might be attributed to the silage pH was lower than 4.4 except for BS, thus there was no massive proliferation of yeasts since the most suitable growth pH of yeasts is 4.4–7.8 (Wang and Wang, 2003). In addition, the population of the yeasts was not very high in raw materials, and the research of Wang et al. (2018) showed the ensiling process had an inhibitory effect on yeast, thus few yeasts were detected after ensiling (< 2.00 log_10_ cfu/g FM).

For any widely used silage in ruminant feeding, the pH is vital to evaluate the silage fermentation quality (Wang et al., 2019). BS reached a pH of 4.75, which was higher than that of legume silage within 300 g/kg DM (Kung and Shaver, 2001), signifying its poor fermentation quality. It is generally accepted that LAB number which is more than 10^5^ cfu/ g FM in fermentation is essential for quality silage (Smith, 1962; Cai et al., 1998). But the number of epiphytic LAB in FB was just 3.67 log_10_ cfu/g FM, and only 3.92 log_10_ cfu/g FM after ensiling in this study, which was far lower than 5.0 log_10_ cfu/g FM, which may lead to a slow formation of LA during fermentation. In addition, the high buffering capacity of faba bean would also slow down the decrease of pH. All of these factors made the final pH difficult to reach the general standard of 4.2. Compared with BS, mixed silage of faba bean with forage wheat or oat supplied more amount of epiphytic LAB in the fermentation process, and the mixed ensiling might produce a synergistic effect on microorganisms (Larsen et al., 2017; Zeng et al., 2020), which was of benefit to the silage fermentation. Therefore, the pH of mixed silage stayed between 4.1 and 4.3, which was lower than 4.75 of FB, and the number of LAB in BO mixed silage was higher than that in BT (Table 4) so its pH was also lower.

As an organic acid produced by LAB, LA is the main organic acid to reduce the pH of silage based on its low dissociation constant (Lima et al., 2010). The LA content of BS was low (19.07 mg/g DM), which was similar to the results of Borreani et al. (2009) and Rinne et al. (2020). The LA content in mixed silage was higher than that of BS, besides the larger amount of LAB, higher WSC content and sugar species after mixing may be contributed (Yan et al., 2019). The LA content of BO mixed silage was higher than that of BT. In addition to the larger number of LAB, the more suitable microflora structure may be one reason why the LA content of BO mixed silage is higher than that of BT. Meanwhile, the LA content was affected by the interaction between species and mixing ratios, resulting in the highest LA content in BO30, but in BT mixed silage, the highest LA content was in BT10. The number of LAB in BO10 and BO30 were the highest in BO mixed silage, so as BT10 in BT mixed silage. The synergistic effect produced by BO mixed silage may be stronger than BT, due to the more suitable microbial flora. Thus, the fermentation of BO30 was more thorough, and the LA content was higher. However, the mechanism of interaction affecting the LA content of mixed silage still needs to be further studied. The PA content in the current study was relatively high (9–10 mg/g DM), but was still basically at the acceptable range (1–10 mg/g DM) of general standard in silage (Agarussi et al., 2019). In addition to propionic acid bacteria, enterobacteria could also metabolize substrates to PA (Urdaneta et al., 1995). Therefore, the high content of PA may be related to the bacterial community.

The content of NH_3_-N in silage was considered relative to CP, since it revealed the extent of proteolysis in silage (Jian et al., 2017). Notably, the loss of CP also increased with the increase of faba bean mixing ratio in mixed silage treatments, although there was no significant difference in NH_3_-N content in the different mixing ratios. It may be the higher proportion of faba bean in the mixed silage, the more the CP content and the number of undesirable bacteria, and thus more protein degradation happened during the fermentation. Oliveira et al. (2017) reported that LAB lower than 10^4^ cfu/g FM in raw materials might increase NH_3_-N content after ensiling. In this study, the NH_3_-N content of FB was a high 8.44% TN, but still within the acceptable range of 10% NH_3_-N/TN (McDonald et al., 1991), indicating that although the epiphytic LAB number was only 3.85 log_10_ cfu/g FM in BS, extensive protein hydrolysis did not occur which might be related to the absence of *Clostridium* (Muck, 2010). As for mixed silage, their NH_3_-N contents were lower than that of BS, suggesting that mixed silage effectively inhibited protein degradation, which might be attributed to the larger amount of LAB and WSC content (Rinne et al., 2020).

### Effects of Mixed Silage of Faba Bean With Forage Wheat or Oat on Bacterial Community

The bacterial community is directly related to the ensiling quality since the ensiling process depends on the interactions of multiple bacteria (Ni et al., 2017). After ensiling, Proteobacteria and Firmicutes dominated the entire bacterial community at the phylum level, and the diversity of bacteria decreased, which is consistent with the result of red clover and napiergrass silage (Dong et al., 2019; Wu et al., 2020). Wayne Polley et al. (2007) reported that the microbial community diversity decreased when the dominant bacteria were abundant, which indicates that Proteobacteria and Firmicutes were the major functional bacteria in silage fermentation.

As a member of Firmicutes, LAB plays an essential role in the process of ensiling, during which it ferments carbohydrates, produces LA, and creates an unsuitable circumstance for spoilage microorganisms (Pot et al., 2014). TS and OS were abundant with *Lactobacillus_lindneri* and *Lactobacillus*, respectively, while BS was abundant with *enterobacter, Serratia*, and *Hafnia_Obesumbacterium* (Figure 4), but lack of LAB, which may account for the poor fermentation of BS. Mixed silage increased the relative abundance of LAB (Figure 2), and in BO mixed silage was higher than that in BT, indicating that the microbial structure after mixing faba bean with oat was possibly more conducive to LAB fermentation. The LA content of BO30 was higher than that of BO10 (55.80 mg/g DM vs 45.42 mg/g DM), but its relative abundance of LAB was slightly lower than that of BO10 (60.7 vs. 61.8%). This may be because the *Lactobacillus_plantarum* was abundant in BO30, and its fermentation was more thorough (Figure 4). In addition, it may also be related to some synergistic effects produced after further mixing of legumes and grasses, which is conducive to fermentation. The relative abundance of *Lactobacillus* in BO10 and BO30 was higher than that of OS, suggesting that the two mixing ratios were beneficial to fermentation, which was also evidenced by their high LA content. However, the LA content of BO30 was higher than that of BO10, this may be due to some synergistic effects with the further mixing of legumes and grasses, which benefit the fermentation process. As members of Enterobacteriaceae, *Serratia* and *Hafnia_Obesumbacterium* were more abundant in BO50 than in BO10 and BO30. During the initial fermentation, they could compete with LAB for available carbohydrates (Östling and Lindgren, 1993), which might inhibit the growth of LAB and finally exert a negative impact on the fermentation quality. Therefore, it may be the reason why the fermentation quality of BO50 was poorer than BO10 and BO30. Although the relative abundance of *Lactobacillus* was higher in OS, the LA content of BO50 was higher than that of OS, which also implied some benefits existing in the mixed silage of legumes and grasses. At the same time, the relative abundance of *Lactobacillus* in BT mixed silage gradually decreased, but *Serratia* was still abundant, which may together lead to a poorer fermentation quality than BO. However, the relative abundance of *Lactobacillus* in BT mixed silage was still higher than that in TS, so the LA content was also higher, which indicated that mixed silage was exactly more conducive to LAB fermentation than sole silage.

Previous research on *Serratia* focused more on medicine (Hejazi and Falkiner, 1997; Mahlen, 2011), and less on silage. The presence of a large amount of *Serratia* and *Hafnia_Obesumbacterium* in both the sole and mixed silage may be related to the fact that the primary effect of *enterobacter* on silage comes mainly under anaerobic conditions (Muck, 2010). Szewzyk et al. (1993) found the growth and survival rate of *Serratia* was the highest under anaerobic conditions, and it could not survive under aerobic and semi-anaerobic conditions, which may be the reason for its increment after ensiling. Duan et al. (2020) found *Serratia* and *Hafnia_Obesumbacterium* in spoiled chicken breast, and these bacteria were effectively inhibited by some antimicrobial substance from *Lactobacillus*. In this study, it is found that *Lactobacillus* also had a similar inhibitory effect. The silage with a higher relative abundance of *Lactobacillus* showed a lower relative abundance of *Serratia* and *Hafnia_Obesumbacterium*, such as BO10 and BO30. Especially when the relative abundance of *Lactobacillus* was over 50%, *Serratia* and *Hafnia_Obesumbacterium* became very few. This may be due to the more LA produced by a higher relative abundance of *Lactobacillus*, which reduced pH and inhibited the growth of *Serratia* and *Hafnia_Obesumbacterium*, suggesting that these two bacteria were acid intolerant. Alternatively, the relatively high abundance of Lactobacillus took advantage of the competition with them for fermentation substrates, resulting in their lower relative abundance. Further research is needed.

### Effect of the Bacterial Community on Fermentation Quality

Silage fermentation is actually a process initiated by microorganisms, which significantly affects the nutritive aspects and fermentation quality of forage through a series of end products (Kung Jr et al., 2018; Xin et al., 2021). The present research showed a significant correlation between bacterial community and fermentation quality, which was similar to the study of McAllister et al. (2018). As the vital functional bacteria of silage fermentation, *Lactobacillus* promoted the accumulation of LA, inhibited the production of NH_3_-N, PA, and BA, and thus improved the quality of silage fermentation. A similar effect of *Lactobacillus* was also found in Italian ryegrass, alfalfa, and king grass fermentation (Yan et al., 2019; Yang et al., 2020; Zi et al., 2021). *Serratia* and *Hafnia_Obesumbacterium*, which belong to Enterobacteriaceae, were negatively correlated to LA content and positively correlated to NH_3_-N content, and these two bacteria could degrade LA and initiate ammonia production through varied deamination reactions, promote LA and protein decomposition, and finally affect the chemical compositions and fermentation quality (Östling and Lindgren, 1993; Wang et al., 2018). Moreover, *Serratia* and *Hafnia_Obesumbacterium* were positively correlated with BA content, indicating that it may be related to the corruption of silage. Amer et al. (2012) reported that the reason for saccharolytic *clostridia* fermenting LA to produce BA may be the insufficient WSC and higher water content of forage in the secondary silage fermentation. In this study, BO mixed silage was about 25% DM, thus it is worth noting that a similar situation may occur in the feeding stage. In conclusion, the bacterial community significantly affected silage quality by affecting the contents of LA, PA, BA, and NH_3_-N, and their effects were bidirectional.

## Conclusion

The study indicated that mixed silage of faba bean with forage wheat or oat had significant effects on chemical compositions and fermentation quality, and at the same time improved the fermentation quality. Mixed silage improved the bacterial community, and more epiphytic LAB led to a relatively high abundance of *Lactobacillus* after ensiling, which increased LA production and reduced pH, so that it inhibited the proliferation of Enterobacteriaceae, reduced the production of PA and NH_3_-N, and finally showed better fermentation quality. The fermentation quality of BO mixed silage was higher than that of BT mixed silage, and the mixing ratio of 3:7 was the best overall, thus BO30 is recommended for the faba bean mixed silage.

## Data Availability Statement

The datasets presented in this study can be found in online repositories. The names of the repository/repositories and accession number(s) can be found at: https://www.ncbi.nlm.nih.gov/, PRJNA778801.

## Author Contributions

HL and TZ: formal analysis, investigation, data curation, and writing original draft, review, and editing. XD, YX, and ZD: formal analysis, investigation, and data curation, YW, BW, and WY: improving the original draft. LH, LL, BK, and DJ: formal analysis and investigation. YY: conceptualization, review and editing, supervision, project administration, and funding acquisition. All authors contributed to the article and approved the submitted version.

## Funding

This study was financially supported by the National Natural Science Foundation of China (grant number 32001401) and Sichuan Science and Technology Department Programs (grant numbers 2020YFN0021, 2021YFH0155, 2021YFN0059, and 2021YFQ0015).

## Conflict of Interest

The authors declare that the research was conducted in the absence of any commercial or financial relationships that could be construed as a potential conflict of interest.

## Publisher's Note

All claims expressed in this article are solely those of the authors and do not necessarily represent those of their affiliated organizations, or those of the publisher, the editors and the reviewers. Any product that may be evaluated in this article, or claim that may be made by its manufacturer, is not guaranteed or endorsed by the publisher.
